# Preliminary efforts in biobanking and plasma biomarkers by the African Dementia Consortium

**DOI:** 10.1002/alz70856_105508

**Published:** 2026-01-07

**Authors:** Jean N Ikanga

**Affiliations:** ^1^ Emory University, Atlanta, GA, USA; University of Kinshasa, Kinshasa, Kinshasa, Congo (The Democratic Republic of the)

## Abstract

**Background:**

The use of biobanking and fluid biomarkers in Western countries has revolutionized Alzheimer's disease (AD) and dementia research. This study presents preliminary efforts in biobanking and plasma biomarkers by the Recruitment and Retention for Alzheimer's Disease Diversity in the Alzheimer's Disease Sequencing Project (READD‐ADSP). The project aims to recruit 5000 African participants (both AD patients and cognitively unimpaired controls) to generate genomic and biomarker data to better characterize AD neurobiology in Africa, focusing on countries that constitute the African Dementia Consortium.

**Methods:**

Blood samples from older adults over 50 years old from more than 10 countries in Sub‐Saharan Africa (SSA) are collected, separated into fractions, and transported to the African Coordinating Centre in Ibadan, Nigeria. There, DNA extraction and long‐term biospecimen storage are carried out. Plasma and DNA aliquots are sent to the John P. Hussman Institute for Human Genomics at the University of Miami for genotyping, whole genome sequencing, and biomarker analysis. Innovative solutions were devised to mitigate challenges encountered so far.

**Results:**

Significant challenges related to blood collection from many participants in SSA arise due to conceptions of blood and the transportation of samples out of Africa. The stepwise creation and development of the READD‐ADSP biobanking network have been guided by global best practices and regulatory standards. Challenges were encountered in the process of establishing the READD‐ADSP biobanking, and home‐grown solutions were developed. Preliminary analyses of plasma AD biomarkers have been conducted, including core AD, non‐specific, and neuroinflammation AD biomarkers. Initial genotyping results show the prevalence of APOE in these populations. Additionally, some associations between these plasma biomarkers with cognitive functions and neuroimaging data have been identified.

**Conclusion:**

The READD‐ADSP biobanking experience demonstrates the feasibility of establishing a successful African biobanking network and offers lessons to researchers in low‐resource settings on how collaborative efforts between the global north and global south enhance cutting‐edge team science to tackle aging‐associated brain disorders in low‐ and middle‐income countries. This network is an important infrastructure to support AD/ADRD research in Africa and highlights the need to build local infrastructure for biomarker and genotyping, genomics, and other omics analyses.

